# Characterisation of Fat Crystal Polymorphism in Cocoa Butter by Time-Domain NMR and DSC Deconvolution

**DOI:** 10.3390/foods10030520

**Published:** 2021-03-02

**Authors:** Arnout Declerck, Veronique Nelis, Sabine Danthine, Koen Dewettinck, Paul Van der Meeren

**Affiliations:** 1 Particle and Interfacial Technology, Faculty of Bioscience Engineering, Ghent University, B-9000 Ghent, Belgium; arnodec@hotmail.com (A.D.); nelis.veronique@gmail.com (V.N.); 2 Food Structure & Function Research Group, Faculty of Bioscience Engineering, Ghent University, B-9000 Ghent, Belgium; Koen.Dewettinck@Ugent.be; 3 Food Science and Formulation, Gembloux Agro-Bio Tech, University of Liege, B-5030 Gembloux, Belgium; sabine.danthine@uliege.be

**Keywords:** fat crystallization, TD-NMR, deconvolution, polymorphism, cocoa butter

## Abstract

The polymorphic state of edible fats is an important quality parameter in fat research as well as in industrial applications. Nowadays, X-ray diffraction (XRD) is the most commonly used method to determine the polymorphic state. However, quantification of the different polymorphic forms present in a sample is not straightforward. Differential Scanning Calorimetry (DSC) is another method which provides information about fat crystallization processes: the different peaks in the DSC spectrum can be coupled to the melting/crystallisation of certain polymorphs. During the last decade, nuclear magnetic resonance (NMR) has been proposed as a method to determine, qualitatively and/or quantitatively, the polymorphic forms present in fat samples. In this work, DSC- and NMR-deconvolution methods were evaluated on their ability to determine the polymorphic state of cocoa butter, with XRD as a reference method. Cocoa butter was subjected to two different temperature profiles, which enforced cocoa butter crystallization in different polymorphic forms. It was found that XRD remains the best method to qualitatively determine the polymorphic state of the fat. Whereas the quantitative NMR and DSC deconvolution results were not fully in line with the XRD results in all cases, NMR deconvolution showed great promise both in a qualitative and quantitative way.

## 1. Introduction

In food science and industry, it is well known that the polymorphic state of fat is an important quality parameter of the lipid phase in the food product. Thus, cocoa butter in chocolate should be crystallised in the β_2_ polymorphic form (which is also indicated as the β_V_ form in the chocolate industry) to obtain the desired snap, gloss and melting range as well as to avoid quality defects due to fat bloom at the chocolate surface [1,2,3,4]. In contrast, small β’ crystals are favoured in margarine as they enable the formation of a good crystal network throughout the continuous lipid phase which is related to good sensory and texture properties [5,6,7].

Fat crystal polymorphism is characterized by the capability of triacylglycerol (TAG) molecules to arrange in different crystal lattice structures yet having an identical TAG composition. The three basic subcell packing structures of TAG acyl chains are a hexagonal, orthorhombic and triclinic subcell, also known as α, β’ and β packing, respectively, of which the thermodynamic stability, the packing density, the melting point and the melting enthalpy increase in consecutive order [8,9,10,11]. Subsequently, these differences in physical properties can be exploited for elucidation of the polymorphic form using various analytical techniques, e.g., Differential Scanning Calorimetry (DSC), nuclear magnetic resonance (NMR), X-ray diffraction (XRD), or infrared spectroscopy. To date, XRD is the standard technique to study fat crystal polymorphism as wide angle X-ray diffraction (WAXD) patterns are a blueprint of the subcell packing in the sample according to Bragg’s diffraction law [12,13]. Furthermore, Dewettinck et al. demonstrated the quantification of α and β’ polymorphs using WAXD intensities of characteristic peaks, provided that pure α and β’ crystal scattering signals of the studied fat are available [1]. However, access to XRD equipment is not straightforward in an (industrial) research & development and/or quality control unit, which is not the case for DSC and low field(LF)-NMR. 

The first studies of fat crystal polymorphism using NMR spectroscopy were conducted by means of complex high field (HF) ^13^C- and ^1^H-NMR techniques [14,15,16]. These methods are not straightforward, require complicated data analysis and are carried out using expensive high field set-ups to acquire high resolution spectra. Nevertheless, these studies pointed out differences in acyl chain mobility between different subcell packing forms of pure TAG, which is an attribute that can be analysed using time-domain relaxometry. Van Duynhoven et al. were the first to exploit the sensitivity of NMR to molecular mobility for differentiation between fat crystal polymorphs using low-cost LF-^1^H-NMR spectrometry [17]. TAGs in α-crystals are subjected to the lowest packing density and experience the highest freedom of movement, and are thus characterised by a slower Free Induction Decay (FID) (or T_2_-relaxation) compared to β’ and β-crystals. Furthermore, β’ and β-crystals can be identified by a ‘beat’ pattern in the FID-curve, which is more outspoken for β than β’-crystals [17,18,19]. Deconvolution of the NMR signal enables the detection of these differences in relaxation behaviour and thus polymorphic form. Different mathematical models have been described in the literature to identify and/or quantify the polymorphic forms [17,18,20,21,22].

In the study of fat crystal polymorphism, DSC endotherms are often analysed to screen the melting, and thus polymorphic, behaviour of a fat, to understand the relationship between these two properties and to identify polymorphic forms in a fat sample based on the characteristic melting point [13,23,24,25,26]. Yet, a confirmation of the different solid phases of the fat by XRD is imperative. So far, DSC melting curves are often not exploited to their full potential as these endotherms also hold quantitative information on the polymorphic crystals, which can be retrieved from the melting enthalpy [27].

As natural fats are complex systems due to their variety of TAG compounds, they may contain a variety of solid phases, including polymorphic forms and pure/mixed crystals, depending on the thermal history [12,28]. Hence, the observed endotherm will be the result of several superimposed thermal processes, which further complicates the interpretation of the melting curves. Deconvolution enables to resolve the resultant melting curve into the individual endothermal contributions [29,30,31,32,33,34], assuming that no exothermal processes, such as recrystallisation or polymorphic transitions, take place. The latter condition is typically fulfilled at high heating rates (e.g., 5 °C/min and higher) [1,13]. Hence, DSC-deconvolution may be used to identify and quantify polymorphic phases in fats as demonstrated by Fessas et al. [35].

The goal of this study was to evaluate both LF-NMR-deconvolution and DSC-deconvolution in their ability to determine the polymorphic state of cocoa butter in a qualitative and preferentially also in a quantitative manner. Cocoa butter was selected because it is a well-studied fat with a known polymorphic behaviour [4,36,37]. The obtained results were compared to results from X-ray diffraction measurements. Hereby, the accuracy as well as the applicability for routine analysis is discussed.

## 2. Materials and Methods

### 2.1. Materials and Temperature Profile

All measurements were performed on West African cocoa butter (pure prime press), which was purchased from Cargill Chocolate Belgium (Mechelen, Belgium). In order to have a diversity of polymorphic states, the fat was subjected to two different time-temperature protocols (Table 1), based on the standard methods AOCS Cd 16b-93 and IUPAC 2.150 for SFC (Solid Fat Content) determination of stabilising and non-stabilising fats by NMR [13]. Stabilising fats (e.g., cocoa butter) are conventionally treated with a tempering procedure which includes a 40 h isothermal period at 26 °C to obtain the most stable β polymorph [13]. It is thus expected to obtain metastable α and β’ cocoa butter crystals using the ‘Non-tempered’ time-temperature protocol and stable β crystals using the ‘Tempered’ time-temperature protocol (Table 1). The measurements were performed at four different temperatures, i.e., 0, 5, 15 and 25 °C. 

### 2.2. XRD

The polymorphic state of the cocoa butter was measured using a D8 Advance Diffractometer (Bruker, Rheinstetten, Germany), which was equipped with an X-ray generator Kristalloflex K780 (Bruker, Rheinstetten, Germany) (λ = 1.54178 Å for Cu K-α, 40 kV, 30 mA). The temperature in the TTK 540 diffractometer chamber (Anton Paar, Graz, Austria) of the device was controlled using a TCU 110 system (Anton Paar, Graz, Austria) which was connected to a water bath. 

The non-tempered samples were mounted directly on a flat plate sample holder. The temperature was initially set to 65 °C, after which the temperature sequence (Table 1) was applied. At the end of every isothermal period, both the small angle X-ray scattering (SAXS)- and WAXD-pattern were registered in the 2θ range 1–13° for long spacing (LS) and 15–27° for short spacing (SS) using a Vantec-1 detector (Bruker, Rheinstetten, Germany). 

The tempered samples were prepared outside the XRD device and were carefully mounted on the sample holder at the end of the tempering step at 26 °C. The sample was further cooled in the XRD device to 0 °C after which the rest of the tempered temperature sequence (Table 1) was applied. The WAXD- and SAXS pattern were collected after every isothermal period in the same way as for the non-tempered samples. The obtained patterns were analysed using EVA Diffrac.suite software (Bruker, Billerica, MA, USA) and Excel 2016 (Microsoft, Redmond, WA, USA).

### 2.3. DSC

#### 2.3.1. Device and Temperature Profile Set-Up

A TA Q1000 DSC (TA Instruments, New Castle, DE, USA) was used to determine the heat flow (expressed in W/g) as a function of temperature (during cooling) and time (during the isothermal period). The device was calibrated using indium (TA Instruments), having a melting enthalpy of 28.57 J/g. In addition to indium (*T_m_* = 156.60 °C), azobenzene (Sigma-Aldrich, Bornem, Belgium) and undecane (Acros Organics, Geel, Belgium), having a melting temperature of 68.50 and −26.00 °C, respectively, were used for temperature calibration. Nitrogen gas was used to purge the system. Each sample (8–13 mg) was sealed hermetically in alodined aluminium pans (TA Instruments) and an empty pan was used as a reference. TA Universal Analysis 2000 version 4.5A was used to determine the offset temperature of melting (*T_m,_*_offset_), melting temperature (*T_m_*) and melting enthalpy ($\Delta H_{m}$). *T_m_* is the temperature at the maximum of the peak, while the offset temperature of melting is defined as the temperature at which the extrapolation of the baseline crosses the extrapolation of the descending part of the peak where the slope was the steepest. The baseline to determine the melting enthalpy was obtained by linear extrapolation of the heat flow at higher temperatures. DSC experiments were conducted in duplicate.

The non-tempered samples were introduced in the DSC and subjected to three temperature cycles. In the first cycle, the sample was equilibrated for 10 min at 65 °C and subsequently cooled at 10 °C/min to 0 °C, where it was kept for 90 min. The sample was heated again to 5 °C at 5 °C/min and kept isothermally at 5 °C for 1 h, after which the sample was reheated to 65 °C (at 5 °C/min). The second cycle is comparable to the rst cycle but includes an isothermal period at 15 °C for 1 hour before heating to 65 °C. Likewise, the third cycle includes two additional, successive isothermal periods (of 1 h) at 15 °C and 25 °C before heating to 65 °C. The melting heat of the different polymorphs (as determined by XRD) is deduced in this manner.

The tempered samples were treated according to Table 1 using water baths and introduced in the DSC cups at the end of the isothermal step at 26 °C. The sample was equilibrated at 26 °C in the DSC and cooled to 0 °C at a rate of 10 °C/min. At 0 °C, the sample was kept isothermal for 90 minutes, after which it was heated at 5 °C/min to one or multiple temperatures, depending on the investigated temperature of interest. Four temperatures (0, 5, 15 and 25 °C) were considered. The sample was kept isothermal for 60 min at all temperatures before the actual investigation temperature and eventually molten while heating to 65 °C. The heating rate was 5 °C/min in all cases.

#### 2.3.2. DSC Deconvolution

The DSC deconvolution method was based on the work of Fessas et al. [35]. Deconvolution of the melting curves was performed using Peakfit 4.12 (Systat Software Inc., San Jose, CA, USA) assuming that the endothermic heat flow as a function of the temperature exists of multiple Gaussian contributions. An initial estimation of the position and the shape of the Gaussian distributions was done manually. Hereby, the peak maximum was set at the T_m_ of the different polymorphs of cocoa butter (Table 2). The initial estimation was then further optimised using the Peakfit software (Systat Software Inc., San Jose, CA, USA). If multiple solutions were possible, only the most simple solution which was physically possible was retained. The melting enthalpy of each polymorph ($\Delta H_{m,polymorph,i}$) was determined by integrating the deconvoluted heat flow belonging to the polymorph (i), as a function of the time (in seconds).

The total melting enthalpy ($\Delta H_{m,total}$) was the sum of all the individual melting enthalpies of the different polymorphs. The percentage of a specific polymorph present was determined using Equation (1).

(1) $$\%\;Polymorph_{i}=\frac{\Delta H_{m,polymorph,i}}{\Delta H_{m,total}}\cdot 100$$

### 2.4. NMR

The NMR analyses were performed using an Oxford Maran Ultra 23.4 MHz NMR-device with a 1.8 cm probe (Oxford Instruments, Abingdon, UK), which was temperature controlled. An FID-CPMG sequence was used. Whereas alternative pulse sequences (e.g., Solid Echo or Magic Sandwich Echo) have been proposed to antify the amount of solids in other fields of research, such as polymer science [42], the FID sequence was selected here as this sequence forms the basis for the official methods for Solid Fat Content analysis in lipid research [43]. In every case, the radiofrequency pulse length was 8 µs, followed by a dead time of 7.5 µs and data acquisition over 100 µs with a sampling time (dwell time) of 0.1 µs. The 1.8 cm diameter tubes were put on top of a 3.0 cm high Teflon spacer. The sample height was 2.0 cm (about 3.0 gram). The number of scans was set to 4 and the receiver gain was fixed at 0.4%. All samples were analysed in triplicate (three distinct tubes per sample).

The results of the FID-CPMG sequence were analysed by four different deconvolution methods. Two qualitative and two quantitative methods were evaluated in their abilities to determine the polymorphic state of the fat. When the method was developed to fit to FID data only, the CPMG part of the FID-CPMG signal was ignored. The deconvolution methods were applied using Matlab R2016b (Mathworks, Natick, MA, USA). The Matlab code of the Trezza and Adam-Berret model is available from the authors. The parameter values of the fitted models can be found in Appendix A Table A1, Table A2 and Table A3.

#### 2.4.1. Bi-Gaussian Model

The physical state of TAGs in cocoa butter may be either solid or liquid. The most simple mathematical function to describe these two distinct phases in a FID curve is a bi-Gaussian function (Equation (2)). The first Gaussian represents the fast (apparent) T_2_-relaxation of solids and the second Gaussian the slow (apparent) T_2_-relaxation of liquid oil. Both Gaussians are characterised by the apparent transverse relaxation time constants, $T_{2,Solid}^{*}$ and $T_{2,Liquid}^{*}$, respectively. This bi-Gaussian model has been previously described for determination of the SFC in bulk fat. Reference is made to Declerck et al. [22] for a detailed description of the fitting procedure using Matlab2015b (Matlab, Mathworks, Natick, MA, USA) and fitting parameters. The $I_{Solid}$ and $I_{Liquid}$. parameter values were restricted to be positive, whereas no restrictions were imposed on the estimated $T_{2,Solid}^{*}$-value has been shown to be highly correlated with the polymorphic state of the fat, it may serve as an indicator of the polymorphic form [22,44]: the lower the $T_{2,Solid}^{*}$-value, the lower the molecular mobility and thus the more stable the polymorphic form.

(2) $$I_{FID,fit}\left(t\right)=I_{Solid}\cdot e^{-0.5\left(\frac{t}{T_{2,\;Solid}^{*}}\right)²}+I_{Liquid}\cdot e^{-0.5\left(\frac{t}{T_{2,Liquid}^{*}}\right)²}$$

#### 2.4.2. Adam-Berret Model

Adam-Berret et al. [21] used an alternative model (Equation (3)) to fit the FID relaxation data. This model consists of a simple exponential function and an Abragamian function, which is the product of a Gaussian and a sinc function [17,19,22]. The model was implemented using Matlab 2016b. A least squares optimisation function was used (*lsqcurvefit*-function) in combination with the ‘trust-region-reflective’-optimisation algorithm to fit the model to the FID data. The parameter values ($I_{Solid}$, $I_{Liquid}$, *A*, $T_{2,Solid}^{*}$ and $T_{2,Liquid}^{*}$) were restricted to be positive.

The parameters of the Abragamian function were used to characterise the polymorphic state of the sample. The second momentum ($M_{2}$) of the Abragamian function may be estimated by Taylor expansion, as expressed by Equation (4) [45,46]. This second momentum is sensitive to the packing of the protons in the sample [46].

(3) $$I_{FID,fit\;}\left(t\right)=I_{Solid}\cdot e^{-0.5\cdot {\left(\frac{t}{T_{2,Solid}^{*}}\right)}^{2}}\cdot \frac{\sin\left(A\cdot t\right)}{A\cdot t}+I_{Liquid}\cdot e^{-\frac{t}{T_{2,Liquid}^{*}}}$$

(4) $$M_{2}={\left(\frac{1}{T_{2,Solid}^{*}}\right)}^{2}+\frac{1}{3}\cdot A^{2}$$

##### 2.4.3. Extended Abragamian (EA) Model

Various researchers mention that the T_2_-relaxation behaviour of α-crystals is well described by a Gaussian function, while the T_2_-relaxation pattern of β’ and β crystals is better approximated by an Abragamian function [17,18,19,22]. Since the difference in T_2_-relaxation decay between β’ and β-polymorphs is not distinguishable [18], the contributions of β’ and β-polymorphs to the relaxation decay are combined and represented by β^(^’^)^ in the following discussion. Hence, addition of an Abragamian function to the simple bi-Gaussian function (Equation (5)) enables to describe the FID of a mixture of liquid oil and solid fat in either the α and/or β^(^’^)^-form. This FID model fit has been previously used to determine the SFC [22]. In this study, however, the signal intensities of the α and β^(^’^)^-crystals and of the liquid oil are used to calculate the relative contribution (RC) of the α and β^(^’^)^-polymorphic forms present in the fat sample by Equation (6). Reference is made to Declerck et al. [22] for a detailed description of the fitting procedure using Matlab2015b (Matlab, Mathworks, Natick, MA, USA) and the fitting parameters.

(5) $$I_{FID,fit}\left(t\right)=I_{\alpha }\cdot e^{-0.5{\left(\frac{t}{T_{2,\alpha }^{*}}\right)}^{2}}+I_{\beta^{\left(\prime \right)}}\cdot e^{-0.5{\left(\frac{t}{T_{2,\beta^{\left(\prime \right)}}^{*}}\right)}^{2}}\cdot \frac{\sin\left(A\cdot t\right)}{A\cdot t}+I_{Liquid}\cdot e^{-0.5{\left(\frac{t}{T_{2,\;Liquid}^{*}}\right)}^{2}}\;\;\;$$

(6) $$\mathrm{RC}-\mathrm{component}\;x\;\left(\%\right)=\frac{I_{x}}{I_{\alpha }+I_{\beta^{\left(\prime \right)}}+I_{Liquid}}\cdot 100$$

##### 2.4.4. Trezza Model

The EA model represents the most simplified fitting model to quantify polymorphic forms. However, natural fats may contain imperfect crystals, impurities and/or amorphous phases. Imperfect crystals have a less stringent packing, a lower packing density and thus a higher molecular mobility [18], while the molecular mobility of amorphous solid phases is higher than crystalline solid phases [47,48,49]. These amorphous solid fractions have an intermediate relaxation behaviour and are further referred to as semisolids. In order to detect and/or to distinguish semisolids from liquid oil, a FID-CPMG sequence has been introduced [18,48]. Trezza et al. [18] proposed a deconvolution model based on multiple Gaussian, exponential and Abragamian functions (Equation (7)) to fit the FID-CPMG data. This multiple function approach yields a continuous distribution of *T*_2_^(^*^)^-times. In Table 3, the parameters defined by Trezza et al. [18] are given. Every part of Equation (7) is linked to the relaxation decay of a certain phase in a fat sample, i.e., liquid oil (characterised by *T*_2*,n*_), semisolid (characterised by *T*_2*,i*_), α-polymorphic crystals (characterised by T*_2*,k*_), and β^(^’^)^-polymorphic crystals (characterised by T*_2,*j*_, *B* and *A*). The *T*_2_^(^*^)^-values of every phase are restricted within a predefined range of values (Table 3). Parameter B was optimised for the obtained FID-CPMG data and fixed at a value of 0.06 µs^−1^. In comparison, Trezza et al. [18] used a value of 0.0665 µs^−1^. Matlab 2016 was used for the implementation of the model. A non-least square optimisation algorithm was used to find an optimal fit. The obtained coefficients after deconvolution were used to determine the total intensity of each phase ($I_{\alpha }$, $I_{\beta^{\left(\prime \right)}}$, $I_{Semisolid}$ and $I_{Liquid}$) using Equations (8)–(11), from which the relative contribution of each polymorph present in the fat sample was estimated in a similar way as shown in Equation (6).

(7) $$I_{FID-CPMG,fit}\left(t\right)=\overset{l1}{\underset{n=1}{\sum }}L_{1}\left(n\right)\cdot e^{-\left(\frac{t}{T_{2,n}}\right)}+\overset{l2}{\underset{i=1}{\sum }}L_{2}\left(i\right)\cdot e^{-\left(\frac{t}{T_{2,i}}\right)}+\overset{g}{\underset{k=1}{\sum }}G\left(k\right)\cdot e^{-0.5{\left(\frac{t}{T_{2,k}^{*}}\right)}^{2}}+\overset{l3}{\underset{j=1}{\sum }}L_{3}\left(j\right)\cdot e^{-\left(\frac{t}{T_{2,j}^{*}}\right)}+\overset{p}{\underset{l=1}{\sum }}P\left(l\right)\cdot e^{-0.5{\left(B\cdot t\right)}^{2}}\cdot \frac{\sin\left(A\cdot t\right)}{A\cdot t}$$

(8) $$I_{liquid}=\overset{l1}{\underset{n=1}{\sum }}L_{1}\left(n\right)$$

(9) $$I_{Semisolid}=\overset{l2}{\underset{i=1}{\sum }}L_{2}\left(i\right)$$

(10) $$I_{\alpha }=\overset{g}{\underset{k=1}{\sum }}G\left(k\right).$$

(11) $$I_{\beta \left(\prime \right)}=\overset{l3}{\underset{j=1}{\sum }}L_{3}\left(j\right)+\overset{p}{\underset{l=1}{\sum }}P\left(l\right)$$

### 2.5. Statistics

A Kruskal-Wallis rank sum test was used to find significant differences between the melting temperature and total melting enthalpy at different tperatures. If a significant difference was found, Dunn’s test was used as a post-hoc method. The statistics were done on a 5% significance level using R 3.2.3 and RStudio (Boston, MA, USA) as a graphical interface.

## 3. Results

### 3.1. XRD

**Figure 1:**
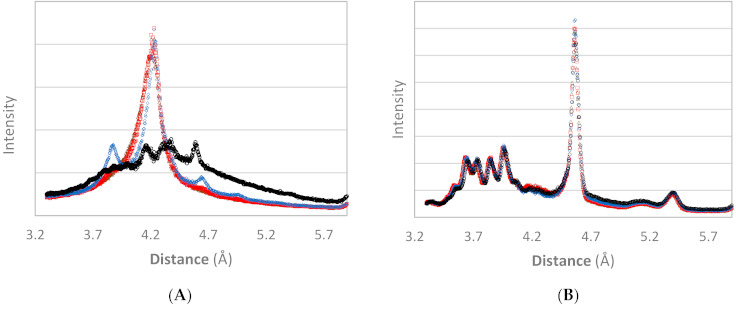
WAXD (wide angle X-ray diffraction) pattern of non-tempered (**A**) and tempered (**B**) cocoa butter obtained after 90 min at 0 °C (brown triangles), 60 min at 5 °C (red squares), 60 min at 15 °C (blue diamonds) and 60 min at 25 °C (black circles).

Comparison of the obtained XRD patterns with published short spacings (SS) and long spacings (LS) of cocoa butter [24,36,37,38,50] allowed identification of the cocoa butter polymorphic form. The WAXD pattern of the non-tempered cocoa butter (Figure 1A) at 0 °C showed only evidence of a hexagonal cross-sectional subcell packing (SS = 4.20 Å). Hence, the crystalline solid fraction is fully attributed to the α phase at 0 °C. At 5 °C, a very strong intensity at 4.20 Å, together with an additional weak SS at 3.87 Å, and a very weak intensity at 4.64 Å, indicated the presence of a (most probably small) fraction of β_2_’-crystals, coexisting with α crystals. At 15 °C, the small peaks related to β_2_’-crystals increased, indicating an increase in the contribution of β_2_’-crystals. The dominant peak around 4.20 Å shifted slightly to higher small spacing values (SS = 4.24 Å) and showed an additional shoulder at 4.34 Å, which might indicate very few β_1_’-crystals. At this stage, it is not unambiguously clear whether the metastable α phase is still present. The WAXD pattern observed at 25 °C shows the coexistence of β_1_’-and β_2_-crystals. Hereby, the absence of a SS around 4.20 Å indicated the melting and/or polymorphic transition of α-and β_2_’-crystals to more stable phases. This is in line with Marangoni and McGauley [25], who also found that the stable β polymorphic form was only observed at higher crystallization temperatures (20–26 °C) and through polymorphic transition from β’ to β.

The WAXD pattern of tempered cocoa butter (Figure 1B) was identical at all experimental temperatures. The very strong intensity around 4.60 Å confirms the dominating presence of β-crystals. The 40 h isothermal step at 26 °C in the tempering protocol (Table 1) was introduced to acquire the most stable β polymorph [13,25,38], which together with the crystal memory effect [24], induced β-phase crystallization upon cooling. The additional peaks around 3.65 Å, 3.74 Å, 3.85 Å, 3.97 Å, 5.1 Å and 5.4 Å indicate β_2_-crystals. Moreover, the peak intensities were comparable at all temperatures, except from 25 °C, where the peak around 4.6 Å decreased slightly. This indicates a comparable amount of crystalline matter (and hence SFC) and thus no significant melting of solid crystals when increasing the incubation temperature from 0 to 25 °C. This observation further supports the dominating presence of the most stable β-crystals: according to Van Malssen et al. [37], the lower temperature of the melting range of the β phase is 29 °C which is higher than the applied temperature. 

### 3.2. DSC

#### 3.2.1. Qualitative Analysis

**Figure 2:**
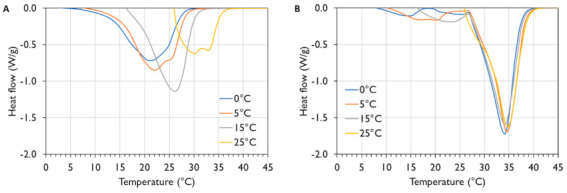
Melting profiles (endo down) of non-tempered (**A**) and tempered (**B**) cocoa butter kept isothermally at 0, 5, 15 or 25 °C.

The melting profile of the non-tempered samples (Figure 2A) indicated that more polymorphic transitions happened when the last isothermal temperature was higher. The peaks shifted more towards higher temperatures. This showed the transition of the unstable α-polymorph towards β’- and β-polymorphs. When the sample was kept at 25 °C, the last peak maximum was found at about 34 °C, which corresponded with the peak maximum found in all tempered samples (Figure 2B). This peak indicated the presence of β_2_-crystals. However, for the non-tempered samples melting peaks were also observed at lower temperatures, which was not the case for the tempered fats. The latter indicated that there was still a substantial amount of less stable polymorphs present.

The above visual observations can also be quantified (Table 4). Although no significant differences were found for the different $T_{m,\mathrm{offset}}$ and $\Delta H_{m,total}\;$of non-tempered cocoa butter samples (*p* = 0.08), mainly because of the limited number of repetitions (i.e., only 2), the offset melting temperature, $T_{m,\mathrm{offset}}$, increased when the cocoa butter was kept at higher temperatures: whereas $T_{m,\mathrm{offset}}$ was about 28 °C if the sample was molten after isothermal storage at 0 °C, it increased to 36 °C if the sample was kept at 25 °C.

This increase indicates the creation of more stable polymorphs. XRD-results revealed that only α-polymorphic crystals were formed at 0 °C, while at higher temperatures, α-crystals were transformed to β’- or β-type crystals. This observation was further sustained by the melting enthalpy. The α-crystals (in the sample held at 0 °C) yielded a melting enthalpy of 89 ± 1 J/g, while at 15 °C, when both α-and β_2_’-crystals were present, the melting enthalpy already rose to about 102 ± 1 J/g. As the amount of crystals in the sample at 15 °C is lower than at 5 °C, whereas the melting enthalpy is higher, this means that β_2_’-crystals have an even higher melting enthalpy relative to α-crystals. Similar results were found when mango almond fat mixtures were analysed [51]: α-crystals had the lowest melting enthalpy, while more stable polymorphs had a higher melting enthalpy, whereas mixtures of different polymorphs had enthalpy values between the enthalpy values of the pure polymorphs [51]. The increasing melting enthalpy and temperature values observed after isothermal incubation at 5 °C and 15 °C, indicated that a larger amount of β_2_’-crystals was formed at higher incubation temperature. At 25 °C, the melting enthalpy was significantly lower (55 ± 3 J/g). At this temperature, all the α-crystals were already molten and thus less crystals were present in the sample, reducing the melting enthalpy.

The tempered cocoa butter samples were all crystallised in a β_2_-polymorphic state according to the XRD-results (Figure 1). This can clearly be observed from the high and similar $T_{m,\mathrm{offset}}$ and total melting enthalpy after the different isothermal periods (Table 5). The $T_{m,\mathrm{offset}}$ was about 39 °C in all cases, while the melting enthalpy was about 140 J/g, indicating the presence of the same polymorphic state in the different samples. At 25 °C the melting enthalpy value was lower (130 ± 1 J/g), which may be ascribed to the partial melting of a fraction of the fat that melts at a lower temperature. For the sake of completeness, it can be mentioned that the $\Delta H_{m,total}$ and $T_{m,\mathrm{offset}}$ did not differ significantly (*p* = 0.31 and 0.08 for $T_{m,\mathrm{offset}}$ and $\Delta H_{m,total}$, respectively) between the different temperatures, which was due to the limited number of replicates (i.e., 2).

#### 3.2.2. Quantitative Analysis

**Figure 3:**
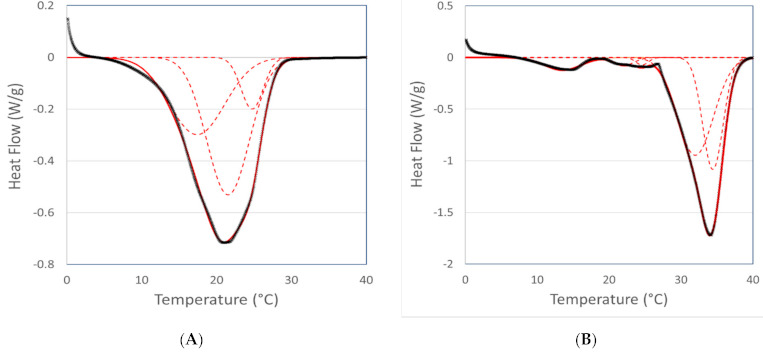
Deconvolution of the DSC melting profiles (endo down) of non-tempered (**A**) and tempered (**B**) cocoa butter incubated at 0 °C: the dotted lines show the different Gaussian contributions to the overall DSC profile (which is shown by the full line).

Several problems were encountered associated with the DSC deconvolution. First of all, DSC peak deconvolution is an ill-posed problem, whereby several solutions (differing in number and relative contribution of the different sub-peaks) are possible, depending on the initial parameter settings. Besides, the fat should be studied by both DSC and XRD under similar conditions in order to be able to assign the correct polymorph to the peaks observed in the DSC profile. Moreover, as illustrated in Table 2, the different polymorphs are characterised by a melting range, rather than a melting temperature, whereby the melting ranges of different polymorphs overlap, which largely complicates peak identification. In fact, without additional information (e.g., from XRD) correct peak assignment is nearly impossible. Due to this complication, the peaks were only assigned to the main crystal polymorphs (i.e., α, β’ or β), without further subdivision. Lastly, the melting enthalpy per gram of β-polymorphs is higher than the melting enthalpy per gram for α-type crystals. This will make that the DSC method overestimates the amount of β-crystals in comparison with the α-crystals, when only considering the relative peak areas. Despite of these drawbacks, a proof of concept is given using the average melting profiles of the tempered and non-tempered cocoa butter. Figure 3 illustrates the deconvolution for the tempered and non-tempered samples incubated at 0 °C.

The $\Delta H_{m,total}$ obtained using DSC deconvolution for both the tempered and non-tempered samples was comparable with the $\Delta H_{m,total}$ obtained from the direct integration of the DSC results. The melting temperature of each individual Gaussian contribution is shown in Table 6 and Table 7. Using Equation (1), the melting enthalpy results were recalculated to relative contributions.

Based on Table 2, the melting temperatures were linked to a given polymorph. Although mainly β_2_-crystals were observed in the XRD spectrum of the tempered samples, the DSC method showed that multiple Gaussian contributions were present. Overall, the trend from less stable to more stable polymorphs was logical for both the tempered and non-tempered fats. At lower storage temperatures, the percentage of α-crystals was higher compared to the higher storage temperatures for the non-tempered fats, while the amount of β’- and β-crystals rose as the isothermal holding temperature was increased. A similar trend towards more stable polymorphs at higher holding temperatures was also observed for the tempered fat.

### 3.3. NMR

#### 3.3.1. Qualitative Analysis

**Figure 4:**
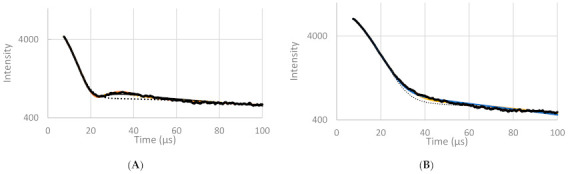
FID (Free Induction Decay) signal and the fits of the different models of tempered (**A**) and non-tempered (**B**) cocoa butter at 0 °C. Black line is the raw signal, orange line is the fit of the Trezza model, dotted grey line is the fit of the bi-Gaussian model, yellow line is the fit of the EA model, blue line is the fit of the Adam-Berret model.

**Figure 5:**
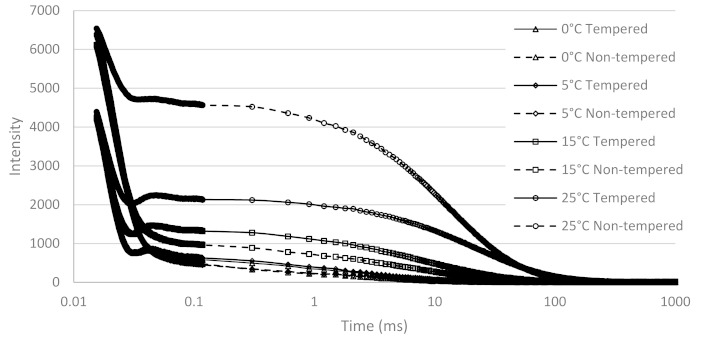
FID-CPMG signal of tempered (full lines) and non-tempered (broken lines) cocoa butter at 0 °C (triangles), 5 °C (diamonds), 15 °C (squares), and 25 °C (circles).

At 0 °C, the α-polymorph was (mainly) obtained in the non-tempered sample and the β-polymorph in the tempered sample according to the XRD-data. The same conclusion can be obtained from the FID signal of tempered (Figure 4A) and non-tempered cocoa butter (Figure 4B) at 0 °C: whereas the typical beat pattern is observed in the tempered sample, a slower FID decay was observed in the non-tempered sample. A similar behaviour was also observed in the FID part of the FID-CPMG curves at other storage temperatures, as shown in Figure 5. In Figure 4, it can also be seen that in the case of the tempered sample, the presence of an Abragamian function (in the Adam-Berret model, the EA model and the model of Trezza) was necessary to fit the local maximum around 30–50 µs. In the latter case, the model fits could hardly be discerned from the experimental data (Figure 4), which was not the case for the bi-Gaussian model.

The $T_{2,Solid}^{*}$-value determined with the bi-Gaussian model of the β-polymorph (6.6–7.6 µs) was lower than the $T_{2,Solid}^{*}$-value of the α-polymorph (11.2–11.9 µs at temperatures below 25 °C) (Table 8). The second magnetic moment $M_{2}$, on the other hand, was higher for the β-polymorph in comparison with the α-polymorph. For completeness, it has to be mentioned that the standard deviation of the $T_{2,Solid}^{*}$- and *M*_2_-values is not shown in Table 8 because their values were 0.25 µs and 0.0003 µs^2^ at the most; the latter values were obtained for the non-tempered sample at 25 °C, which was due to its much lower solid fat content. In principle, the $M_{2}$- and $T_{2,Solid}^{*}$-value could be used to quantify the relative contribution of a certain polymorph. In the case of the $T_{2,Solid}^{*}$-value, two distinct values for the pure α- and β^(^’^)^-polymorph could be obtained. Intermediate values indicate that both polymorphs are present. The contribution of both polymorphs might be estimated, considering the experimentally determined $T_{2,Solid}^{*}$-value as a weighted average of the $T_{2,Solid}^{*}$-values of both polymorphs. A similar reasoning can be done for the $M_{2}$-values. 

At 5 °C and 15 °C, a mixture of α and β’ was observed using XRD in the non-tempered cocoa butter. This conclusion was further sustained by the $T_{2,Solid}^{*}$ and $M_{2}$ values. Both parameters had intermediate values between those of pure α- and β^(^’^)^-crystals. Assuming that the $M_{2}$ value of α-crystals and β^(^’^)^-crystals is 0.0073 and 0.0157 µs^2^ resp., the experimental values for the non-tempered samples at 5 °C and 15 °C (0.0074 and 0.0084 µs^2^, respectively) indicate a relative contribution of 99% and 87% for α-crystals at 5 °C and 15 °C, resp. As the $T_{2,Solid}^{*}$-value is temperature dependent [52], it is hard to decide whether the difference in $T_{2,Solid}^{*}$-values at different temperatures is due to the temperature as such or rather due to a shift in polymorphs. This is nicely illustrated by the decreasing $T_{2,Solid}^{*}$-values for the tempered samples at increasing incubation temperatures, despite of their highly similar XRD profiles. Hence, $T_{2,Solid}^{*}$-values can only be considered as a good indicator for the presence of polymorphs for isothermal measurements. Kaufmann et al. [53] used a similar parameter to determine the polymorphic transitions in milk fat in combination with rapeseed oil and shear. They found that high shear rates during isothermal conditions decreased the amount of α-polymorphs and accelerated the polymorphic transition towards β’-crystals. The second moment, as proposed by Adam-Berret, is, however, much less temperature-dependent as can be clearly seen from the similar values for all tempered samples studied.

#### 3.3.2. Quantitative Analysis

The quantitative models showed a trend towards the expected polymorph. Whereas no α-polymorph was observed in the tempered sample by XRD, the results of both models (EA and Trezza) indicated that there were some α-crystals present (Table 8). On the other hand, the non-tempered samples were expected to only contain α-polymorphs at 0 °C, whereas both models still indicated the presence of β^(^’^)^-polymorphs: the Trezza model and EA model determined the amount of β^(^’^)^-polymorphs to be around 22% and 16%, respectively (Table 8).

The EA model did not give a consistent trend in the percentage of α crystals. Whereas a lower amount of α crystals is expected as the temperature rises, the estimated percentage at 5 °C was 21.9 ± 0.3% and increased to 29.3 ± 1.2% at 15 °C. This trend was not supported by XRD measurements and seems hard to explain as α-crystals are thermodynamically less stable than β-polymorphs. The model of Trezza showed a more clear trend in the percentages of the different polymorphs. The percentage of α-polymorphs got lower when the temperature rose and was eventually about 0% at 25 °C in both tempered and non-tempered cases. Whereas these quantitative values may not reflect the actual polymorphic composition of the fat, still they seem promising as a (rough) estimate of the composition, as well as for trend analysis.

The differences in results obtained using the EA and Trezza model had multiple causes. The main difference is related to the used pulse sequence, which was a simple FID in the case of the EA model, and a combined FID-CPMG sequence in the case of the Trezza model. The additional CPMG-sequence mainly enables a better characterisation of the (slowly decaying) liquid phase. A second difference is that the Trezza model analysed a predefined range of $T_{2}$-values, which were linked to specific equations (Gaussian, Lorentzian or Abragamian), while the EA model only uses one $T_{2}$-value for each polymorph. In addition, the Trezza model also took semisolid phases into account, which is not the case for the EA model. 

From the results in Table 8, it can be concluded that the Trezza model is superior in the case of quantification. If only a qualitative investigation of the polymorphs is necessary, the $T_{2,Solid}^{*}$ can be used as an indicator.

For most of the 8 experimental conditions (i.e., 4 temperatures, either without or with tempering), the FID or FID-CPMG deconvolution predicts the most abundant polymorph, whereby the conclusions drawn from the Extended Abragamian and from the Trezza model are largely in agreement.

The only exception is the non-tempered sample at 25 °C, for which the Extended Abragamian model predicts an excess of α-crystals, whereas XRD indicates mainly β and/or β’ crystals. The fact that this sample has by far the lowest solid fat content (which is less than 50%) provides a logical explanation. Figure 5 indeed clearly shows that the initial fast decaying part of the FID-CPMG curves (which contains the information related to the solid fraction) was much less pronounced (relative to the slower decaying part, due to the liquid fraction), which makes accurate parameter estimation of the solid fraction much less obvious. The Trezza model, on the other hand, enabled a correct determination of the main polymorph, even at these low solid fact content values. Considering the non-tempered cocoa butter at 15 °C, both the EA and the Trezza model predict that mainly α-crystals are present. XRD indicated that this sample contained both α and β^(^’^)^ crystals. However, from XRD, it is hard to make a direct quantitative estimation. Besides, DSC also indicated an excess of α over β^(^’^)^ crystals.

## 4. Discussion

The polymorphs present in cocoa butter, submitted to two different temperature programs, were identified by means of XRD-, NMR- and DSC-measurements. The XRD results showed that while the tempered sample was in a stable β_2_-polymorph, the polymorphic state of the non-tempered fat changed from α- to β_2_’- and eventually to β_2_-crystals with increasing temperature. 

DSC is an excellent way to get quantitative information (i.e., melting temperatures and enthalpies) about polymorphic transitions. DSC deconvolution as a quantitative way to determine the relative contribution of different polymorphs suffers from the fact that the results obtained by this method depend to some extent on the (user-defined) initial conditions. Moreover, the different components obtained by deconvolution must be linked to a certain polymorph, which cannot be unambiguously done. Therefore, quantitative results obtained from DSC should be interpreted with much care although a logical trend was found as confirmed by XRD measurements.

By curve fitting of the experimental FID signal obtained by TD-NMR, parameters were derived which could be related to the polymorphic state of the fat. Thus, the value of the second (magnetic) moment ($M_{2}$) was lower in the presence of α-crystals as compared to β^(^’^)^, while the $T_{2,Solid}^{*}$-value was higher. As the $T_{2,Solid}^{*}$-value is also influenced by the temperature, it follows that $M_{2}$ showed the best correlation with the polymorphic state. In a further attempt to quantify the amount of polymorphs present by FID or FID-CPMG deconvolution, both the Extended Abragamian (EA) and the Trezza model were used. Hereby, the Trezza model gave values which were more in (qualitative) correspondence with the XRD measurements compared to the EA model. However, the obtained values should be used as an indication of the polymorphs present, rather than as the exact analytical composition. In this respect, the absence of a standard with known polymorphic composition makes it difficult to test the accuracy of newly introduced methods. Comparing the different techniques, it is also important to mention that FID-measurements require only a limited sample preparation, as well as a short measurement time, which opens interesting perspectives for the investigation of fast polymorphic transitions using TD-NMR.

## 5. Conclusions

From the results of this study, it can be concluded that XRD will remain the standard method to determine unambiguously which polymorphs are present in fat. As, however, XRD equipment is mostly not available in a standard fat research lab, whereas TD-NMR frequently is, this means that the experimentally simpler approach using deconvolution of TD-NMR or DSC data may open interesting perspectives for polymorphic characterisation in routine labs. In fact, both XRD and DSC require complex additional experiments for polymorph identification, whereas the investigated TD-NMR deconvolution approaches only involve a more sophisticated analysis of the TD-NMR data which are available anyway from routine SFC measurements. Whereas this deconvolution approach is mathematically more challenging, this hurdle can be overcome by integrating it as a user-friendly module within the software of these instruments. 

## Figures and Tables

**Table 1:** Applied temperature profile for tempered and non-tempered cocoa butter.

Tempered		Non-Tempered	
T (°C)	Time (min)	T (°C)	Time (min)
100	15	100	15
65	5	65	5
0	90	0	90
26	2400	-	-
0	90	-	-
5	60	5	60
15	60	15	60
25	60	25	60

**Table 2:** Ranges of melting points of the different polymorphs of cocoa butter [9,12,23,37,38,39,40,41].

Polymorph	Melting Temperature (°C)
sub-α	13.0–18.0
α	17.1–24.0
β_2_’	22.4–28.0
β_1_’	21–33.0
β_2_	30.0–34.5
β_1_	33.5–36.3

**Table 3:** Parameter values used for the Trezza model (Equation (7)) based on Trezza et al. [18].

Parameter	Range of Investigated Values	Spacing	Number of Points
$T_{2,n}$	2 ms–1.2 s	logarithmic	$l_{1}$ = 100
$T_{2,i}$	50 µs–2 ms	logarithmic	$l_{2}$ = 100
$T_{2,j}^{*}$	17 µs–45 µs	logarithmic	$l_{3}$ = 100
$T_{2,k}^{*}$	11 µs–13 µs	linear	g = 20
A	0.145 µs^−1^–0.190 µs^−1^	linear	p = 40

*T*_2_,*_n_*: liquid oil; *T*_2_,*_i_*: semisolid; T*_2_,*_k_*: α-polymorphic crystals; T*_2,*j*_ and *A*: β(’)-polymorphic crystals.

**Table 4:** The offset temperature of melting and total melting enthalpy of non-tempered cocoa butter kept at several temperatures, determined via DSC (Differential Scanning Calorimetry). The polymorphic type was obtained from XRD-data.

T (°C)	Polymorph	*T_m_*_,offset_ (°C)	Δ*H_m,total_* (J/g)
0	α	28.1 ± 0.2	89.2 ± 0.9
5	α + (β_2_’)	28.8 ± 0.3	96.1 ± 2.3
15	(α) + β_2_’	30.9 ± 0.3	101.8 ± 0.6
25	β_1_’+ β_2_	36.4 ± 0.1	54.7 ± 2.6

**Table 5:** The offset temperature of melting and total melting enthalpy obtained with DSC of tempered cocoa butter kept at several temperatures. The polymorphic type was obtained from XRD-data.

T (°C)	Polymorph	*T_m,_*_offset_ (°C)	Δ*H_m,total_* (J/g)
0	β_2_	38.4 ± 0.6	140.3 ± 1.7
5	β_2_	39.1 ± 0.4	147.6 ± 1.2
15	β_2_	39.1 ± 0.3	134.1 ± 5.3
25	β_2_	39.9 ± 0.7	129.5 ± 0.9

**Table 6:** Melting temperature and enthalpy of different polymorphs, as well as relative contribution (RC) of the total melting enthalpy, obtained by deconvolution of the DSC results of tempered cocoa butter.

	0 °C			5 °C			15 °C			25 °C		
	T_m_ (°C)	Δ*H_m,poly_* (J/g)	RC (%)	T_m_ (°C)	Δ*H_m,poly_* (J/g)	RC (%)	T_m_ (°C)	Δ*H_m,poly_* (J/g)	RC (%)	T_m_ (°C)	Δ*H_m,poly_* (J/g)	RC (%)
sub-α	13.6	9.3	6.8	15.3	10.9	7.5						
α	22.1	3.3	2.4	20.2	12.5	8.6	19.8	4.8	3.6			
β’	24.6	1.9	1.4				23.5	11.1	8.4	28.0	8.9	7.1
β	32.0	78.2	57.5	30.5	34.0	23.3	30.2	31.3	23.6	30.5	22.9	18.3
β	34.4	43.5	31.9	34.8	88.5	60.7	34.2	85.2	64.4	34.7	93.4	74.6
SUM		136.1	100		146.0	100		132.4	100		125.3	100

**Table 7:** Melting temperature and enthalpy of different polymorphs, as well as relative contribution (RC) of the total melting enthalpy, obtained by deconvolution of the DSC results of non-tempered cocoa butter.

	0 °C			5 °C			15 °C			25 °C		
	T_m_ (°C)	Δ*H_m,poly_* (J/g)	RC (%)	T_m_ (°C)	Δ*H_m,poly_* (J/g)	RC (%)	T_m_ (°C)	Δ*H_m,poly_* (J/g)	RC (%)	T_m_ (°C)	Δ*H_m,poly_* (J/g)	RC (%)
α	17.4	34.2	39.0	16.6	12.5	13.1	19.7	8.5	8.3			
α	21.5	45.1	51.5	22.0	75.1	78.4	23.6	51.1	49.5			
β’	24.8	8.3	9.4	25.9	8.2	8.5	27.0	43.7	42.3	27.5	8.6	15.7
β’										29.6	20.8	38.0
β										32.7	25.3	46.2
SUM		87.6	100		95.8	100		103.4	100		54.7	100

**Table 8:** $T_{2,Solid}^{*}$ (based on a bi-Gaussian fit), second momentum (*M*_2_) and percentage of α and β^(^’^)^ polymorphs (of the total crystalline phase) determined by the Extended Abragamian model and the Trezza model of tempered and non-tempered cocoa butter. For comparison, the main polymorphs as detected by XRD are indicated, whereby polymorphs between parentheses indicate that they are present in lower quantities.

T (°C)	Tempered	Main Polymorph	$T_{2,Solid}^{*}\;(µs)$	*M*_2_ (µs^2^)	Extended Abragamian		Trezza Model	
					RC-α (%)	RC-β^(’)^ (%)	RC-α (%)	RC-β^(’)^ (%)
0	Yes	β_2_	7.63	0.0157	22.3 ± 0.4	65.2 ± 0.5	6.3 ± 1.1	82.1 ± 1.2
	No	α	11.92	0.0073	74.9 ± 0.3	16.0 ± 0.2	70.6 ± 2.2	22.3 ± 2.4
5	Yes	β_2_	7.51	0.0157	20.9 ± 0.3	65.8 ± 0.3	4.8 ± 0.8	82.9 ± 1.0
	No	α + (β_2_’)	11.81	0.0074	74.1 ± 0.4	17.5 ± 0.4	68.9 ± 0.5	24.4 ± 0.5
15	Yes	β_2_	6.78	0.0160	29.3 ± 1.2	46.5 ± 1.0	0.0 ± 0.0	75.4 ± 0.3
	No	(α) + β_2_’	11.18	0.0084	60.3 ± 0.8	25.0 ± 0.4	54.3 ± 0.7	32.9 ± 0.4
25	Yes	β_2_	6.55	0.0160	12.9 ± 1.0	48.2 ± 1.3	0.0 ± 0.0	60.6 ± 1.3
	No	β_2_ + β_1_’	8.19	0.0142	25.1 ± 8.9	11.7 ± 11.1	2.9 ± 3.5	32.7 ± 6.7

## Data Availability

Data is contained within the article or Appendix A.

## Appendix A

Estimated parameter values of the fit of the Adam-Berret model (Table A1), the Bi-Gaussian model (Table A2), and the extended Abragamian (EA) model (Table A3) to the experimentally determined FID profiles of tempered and non-tempered cocoa butter at 0, 5, 15 and 25 °C.

**Table A1:** Estimated parameter values of the fit of the Adam-Berret model to the experimentally determined FID profiles of tempered and non-tempered cocoa butter at 0, 5, 15 and 25 °C.

T (°C)	Tempered	$I_{Solid}\;(-)$	$T_{2,\;Solid}^{*}\;(µs)$	A (µs^−1^)	$I_{Liquid}\;(-)$	$T_{2,Liquid}^{*}\;(µs)$
0	Yes	5420 ± 7	11.35 ± 0.02	0.154 ± 0.001	968 ± 7	189 ± 4
	No	6717 ± 26	11.67 ± 0.02	0.002 ± 0.001	1011 ± 15	127 ± 3
5	Yes	5287 ± 17	11.63 ± 0.10	0.158 ± 0.001	984 ± 4	222 ± 5
	No	6686 ± 41	11.59 ± 0.01	0.003 ± 0.001	912 ± 3	138 ± 2
15	Yes	4357 ± 7	13.68 ± 0.09	0.179 ± 0.001	1508 ± 12	779 ± 28
	No	5982 ± 55	10.93 ± 0.07	0.005 ± 0.001	1334 ± 31	290 ± 5
25	Yes	3385 ± 24	14.82 ± 0.10	0.185 ± 0.001	2268 ± 20	1757 ± 16
	No	2677 ± 192	11.26 ± 0.51	0.136 ± 0.011	4791 ± 244	2159 ± 145

**Table A2:** Estimated parameter values of the fit of the Bi-Gaussian model to the experimentally determined FID profiles of tempered and non-tempered cocoa butter at 0, 5, 15 and 25 °C.

T (°C)	Tempered	R² (−)	$I_{Solid}\;(-)$	$T_{2,\;Solid}^{*}\;(µs)$	$I_{Liquid}\;(-)$	*T*_2,*Liquid*_* (µs)
0	Yes	0.9961 ± 0.0003	5933 ± 10	7.63 ± 0.00	772 ± 3	131 ± 1
	No	0.9987 ± 0.0000	6934 ± 32	11.92 ± 0.02	722 ± 6	107 ± 2
5	Yes	0.9955 ± 0.0001	5861 ± 20	7.51 ± 0.02	808 ± 2	144 ± 2
	No	0.9986 ± 0.0001	6885 ± 39	11.81 ± 0.01	661 ± 1	116 ± 1
15	Yes	0.9817 ± 0.0005	5381 ± 8	6.78 ± 0.01	1431 ± 9	260 ± 7
	No	0.9971 ± 0.0000	6147 ± 57	11.18 ± 0.07	1118 ± 31	183 ± 1
25	Yes	0.9662 ± 0.0018	4392 ± 23	6.55 ± 0.02	2226 ± 17	357 ± 5
	No	0.9963 ± 0.0005	2850 ± 208	8.19 ± 0.251	4703 ± 247	428 ± 9

**Table A3:** Estimated parameter values of the fit of the Extended Abragamian model to the experimentally determined FID profiles of tempered and non-tempered cocoa butter at 0, 5, 15 and 25 °C; the value of $T_{2,\;\beta^{\left(\prime \right)}}^{*}$ was fixed at 15.81 µs, whereas A was fixed at 0.2 µs^−1^.

T (°C)	Tempered	R² (−)	$I_{\alpha }\;(-)$	T_2,α_* (µs)	$I_{Liquid}\;(-)$	*T*_2,*Liquid*_* (µs)	$I_{\beta \left(\prime \right)}\;(-)$
0	Yes	0.9986 ± 0.0002	1379 ± 26	13.37 ± 0.14	772 ± 3	131 ± 1	4038 ± 34
	No	0.9996 ± 0.0000	5939 ± 28	12.95 ± 0.02	722 ± 6	107 ± 1	1272 ± 21
5	Yes	0.9985 ± 0.0001	1275 ± 19	13.25 ± 0.20	808 ± 2	144 ± 2	4007 ± 17
	No	0.9997 ± 0.0000	5802 ± 56	12.93 ± 0.02	661 ± 1	116 ± 1	1372 ± 27
15	Yes	0.9981 ± 0.0001	1732 ± 68	8.42 ± 0.06	1431 ± 9	260 ± 7	2747 ± 59
	No	0.9995 ± 0.0000	4583 ± 81	13.02 ± 0.04	1118 ± 31	183 ± 1	1898 ± 18
25	Yes	0.9971 ± 0.0003	741 ± 58	9.09 ± 0.12	2226 ± 17	357 ± 5	2756 ± 71
	No	0.9970 ± 0.0011	1867 ± 676	9.72 ± 1.71	4703 ± 247	428 ± 9	865 ± 813
